# Correlations among nicotine dependence, health-related quality of life, and depression in current smokers: a cross-sectional study with a mediation model

**DOI:** 10.3389/fpsyt.2024.1455918

**Published:** 2024-08-27

**Authors:** Huali Xiong, Fengxun Ma, Dayi Tang, Daiqiang Liu

**Affiliations:** ^1^ Department of Public Health, Health Commission of Rongchang District, Chongqing, China; ^2^ Center for Mental Health of Rongchang District, Chongqing, China; ^3^ Department of Public Health, The People’s Hospital of Rongchang District, Chongqing, China; ^4^ First Clinical College, Mudanjiang Medical College, Mudanjiang, Heilongjiang, China; ^5^ Department of Hospital Information, The People’s Hospital of Rongchang District, Chongqing, China

**Keywords:** nicotine dependence, health-related quality of life, depression, mediator, current smokers

## Abstract

**Background:**

Although the negative impact of smoking and health-related quality of life (HRQoL) on depression has been confirmed in various studies, There has been little exploration of how HRQoL mediates the relationship between smoking and depression. The purpose of the current study was to examine the relationship between smoking and depression in the Chinese current smokers with nicotine dependence and the mediating role of HRQoL.

**Methods:**

A cross-sectional study named “Psychology and Behavior Investigation of Chinese Residents” was conducted from July 10 to September 15, 2021 in China. Nicotine dependence, HRQoL and depression were measured by Fagerstrom Test for Nicotine Dependence (FTND), the European Five Dimensional Five Level Health scale (EQ-5D-5L) and the 9-item Patient Health Questionnaire (PHQ-9) respectively. Information on age, gender, place of residence, household registration, education level, marital status, employment status, average family monthly income, drinking frequency, living status, BMI, multiple chronic conditions were also collected. Pearson’s correlation test and logistic regression analysis were conducted to explore the association between nicotine dependence, HRQoL and depression and a mediation analysis was applied to explore the mediating effect of the HRQoL on this relationship.

**Results:**

A total of 1,381 current smokers were included in the study. The participants showed a moderate level of nicotine dependence with a mean of 1.36(SD=1.50), a relatively high level of HRQoL scores (Mean=0.94, SD=0.13), and a depression score with a mean of 6.48(SD=6.09). Approximately 22.74% (314/1,381) of the participants were considered to indicate depression. In the univariable regression model, it was found that nicotine dependence was positively associated with depression (OR:1.094, 95%CI: 1.008-1.187), while HRQoL was negatively associated with depression (OR:0.011, 95%CI: 0.004-0.033). In the multivariable regression model, HRQoL was still notably associated with depression (OR:0.008, 95%CI: 0.002-0.027), however, the positive association was not observed between nicotine dependence and depression. The Pearson’s correlation test demonstrated that nicotine dependence was negatively correlated with HRQoL(r_s_= -0.147, *P*<0.001) and HRQoL was negatively correlated with depression(r_s_= -0.275, *P*<0.001). In contrast, nicotine dependence was positively correlated with depression(rs= 0.136, P<0.001). Mediation analysis found that HRQoL moderated the relationship between nicotine dependence and depression with a mediating effect of 26.49%.

**Conclusions:**

The findings support that nicotine dependence is positively associated with depression and HRQoL is negatively associated with depression in current smokers. HRQoL mediated the relationship between nicotine dependence and depression. The well-established imperative interventions aimed at promoting smoking cessation and improving quality of life may benefit for alleviation of depression in current smokers.

## Introduction

Depression is the most common mental disorder, with a lifetime prevalence rate of about 11-15% (1). Previous studies have demonstrated that age (2), gender and living alone (3), chronic diseases (2), cigarette smoking (4), impaired essential ability of daily living (5) were associated with depression. However, in some areas of China, the stigma of depression prevents patients from receiving diagnosis or treatment (6).

Previous literature suggests a bidirectional relationship between smoking and depression. Some studies have demonstrated that smoking was associated with a higher risk of depression compared with never smoker (7–10), while individuals with depressive symptoms are more likely to engage in smoking behavior (11). This bidirectional and complex relationship forces us to ponder whether nicotine dependence is more linked to depression than smoking behavior. Tobacco/nicotine dependence is classified as a mental and behavioral disorder (12). It is estimated that there are 54 million people aged 40 years and older with severe tobacco dependence in China (13). A Egyptian study found a high correlation between nicotine dependence and depression (14) and studies have emphasized that smoking/nicotine dependence is not only correlated with depression but also leads to respiratory symptoms (15), fatigue, frailty and cognitive decline, ultimately worsening an individual’s health-related quality of life (HRQoL) (16).

The relationship between depression and HRQoL has been observed in older population (17, 18) and individuals with diabetes (19), malnutrition (18), systemic lupus erythematosus (20), adult epilepsy (21). However, individuals with medical conditions, such as friedreich ataxia (22), were found to have their health-related quality of life impacted by the development of depressive symptoms. Similarly, patients with Wilson’s disease (23) exhibited a higher risk of depression. Therefore, evidence in seeking for the association between HRQoL and depression is essential in general population.

Further research under these mechanisms provides new insights into how the independent variable affects the dependent variable. In the current study, we hypothesized that HRQoL mediates the association between nicotine dependence and depression for the following reasons. First, The association between smoking and depression remains controversial because some studies found no link between smoking and depression (24). Second, a previous study found that non-smokers and former smokers have higher HRQoL scores than the current smokers (25). In addition, nicotine dependence was found to be negatively associated with lower HRQoL scores (26), and a poor HRQoL score was negatively associated with depression (27). Few studies have elaborated on HRQoL mediating the relationship between smoking and depression in low and middle income countries, especially in China, which is the world’s largest producer and consumer of tobacco, accounting for one-third of the worlds smoking population (28). Accordingly, it is of great significance to clarify whether HRQoL serves as a mediating role influencing the relationship between nicotine dependence and depression.

Existing studies have revealed the association between nicotine dependence, health-related quality of life, and depression in general populations, while scant attention has been given to the interactions among them in the specific population. It is essential to determine the relationship between nicotine dependence and depression with a large sample size and whether HRQoL may explain this association. Therefore, the aim of current study was to elucidate twofold (1): to explore the relationship between nicotine dependence, HRQoL and depression based on data from community-dwelling smoking adults in China; and (2) to explore whether HRQoL mediates the relationship between nicotine dependence and depression.

## Methods

### Study design and participants

All data were obtained from the “Psychology and Behavior Investigation of Chinese Residents (RBICR) 2021” in China, which was a large-sample, multi-center, replicated cross-sectional study co-sponsored by Peking University. From July 10 to Sep 15, 2021, a multistage random sampling method was conducted in the current study. Firstly, 31 provincial capitals cities including 5 autonomous regions and 4 municipalitie (including Beijng, Tianjin, Shanghai and Chongqing) were directly included. Secondly, 2-6 prefecture-level cities were selected by using a random number table and at last, a total of 120 cities (excluding Hong Kong, Macao, and Taiwan) were included. In order to achieve a representative population distribution, participants of each city were selected using quota sampling (quota attributes such as gender, age, and urban-rural distribution). Consequently, the population distribution of the obtained samples (per 100 people) basically matching the demographic characteristics of the results of the “Seventh National Census in 2021”. This study had been approved by the Medical Ethics Committee of Health Commission of Rongchang District, Chongqing (No. Rcwjw2024018). More details about the study design can be found in the published literature (2, 29–31).

In the current study, only 1,381 cases were included in the analysis, the inclusion and exclusion criteria of the study participants were as follows.

Inclusion criteria: (1) age >18 years; (2) current smokers; (3) participants who voluntarily participate in the current study and complete the informed consent form; (4) participants who complete the questionnaire by themselves or with the help of the investigator;(5) participants who can understand the meaning of each item in the questionnaire.

**Figure 1:**
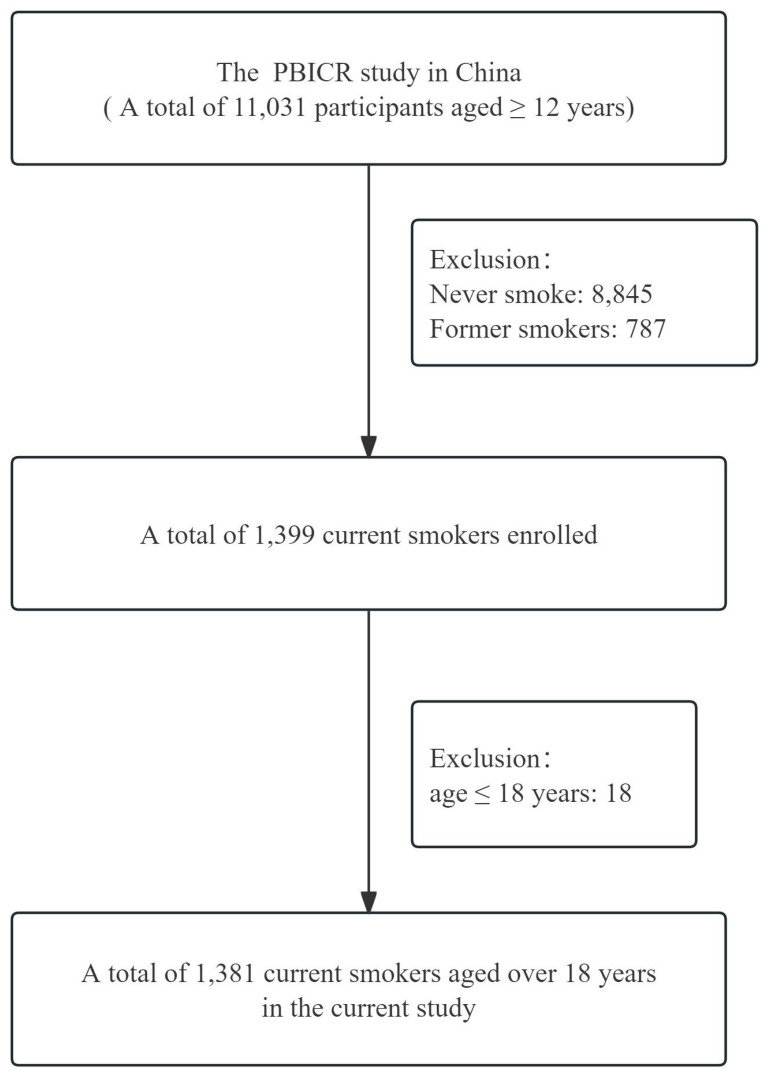
The selection process of study participants.

Exclusion criteria: (1) participants with limited mobility, delirium or mental abnormality; (2) participants who were in other similar research projects; (3) participants unwilling to cooperate with the instructions of the study group. As shown in **Figure 1**, the selection process for study participants was conducted.

### Data collection

With the assistance “Questionnaire Star (https://www.wjx.cn/)”, which was widely used for online questionnaire survey of data collection procedure in China, the survey was conducted from July 10th to Sep 15th, 2021. Investigators or investigation team (less than 10 people) were recruited from local universities. The survey personnel were well trained in sampling methods, research tools and quality control of the study. Prior to the start of the formal survey, a simulated survey was organized to guarantee the quality of investigations; surveyors were considered qualified and competent to participate in the study only if they complied with trained survey procedures (90%). The investigators administered the questionnaire to the participants one by one and face to face. Before the data collection process, participants were asked to click on a link to indicate their informed consent and confirm they understood the survey’s purpose. Participants were assigned a questionnaire number to ensure the uniqueness of the electronic questionnaire. If the participants were capable of thinking but unable to complete the questionnaire on his/her own, the investigator filled out the questionnaire based on the questions he/she answered. A total of 11,709 questionnaires were distributed, and 11,031 valid questionnaires were returned, resulting in 94.2% response rate.

### Measures

#### Nicotine dependence

Two items in the Fagerstrom Test for Nicotine Dependence (FTND) were used to elevate the current smoker’s nicotine dependence level. The first item assessed “How soon after waking up in the morning the participant smoked their first cigarette?” with response options of “>60 min” or “31-60 min” or “6-30 min” or “≤5 min”. The second item asked “How many cigarettes the participant smoked each day?” with response options of “≤10 cigarettes/day” or “11-20 cigarettes/day” or “21-30 cigarettes/day” or “≥31 cigarettes/day”. The scale has good reliability and validity (32, 33). Each response was scored from 0 to 3 depending on the degree of severity (0 =>60 min, 1 = 31-60 min, 2 = 6-30 min, 3=≤5 min;0=≤10 cigarettes/day, 1 = 11-20 cigarettes/day, 2 = 21-30 cigarettes/day, 3=≥31 cigarettes/day). The total score of the two items was 6 points. A higher scores indicates greater nicotine dependence of participants, and a score of ≥4 indicated that severe nicotine dependence might exist (34) and score of 0-1 was defined as light nicotine dependence and score of 2-3 was defined as moderate nicotine dependence.

#### Health-related quality of life

HRQoL was measured with the European Five-Dimensional Five-level Health Scale (EQ-5D-5L) instruments (35), which allows for better differentiation of respondents’ health status and objectify the subjective experience (36). It is commonly used for estimating HRQoL. HRQoL refers to a multi-dimensional indicator for estimating physical state, mental function, social competence, and overall personal condition (37), which could reflect the impact of diseases, physical or mental impairment on an individual’s overall quality of health. The EQ-5D-5L instrument consists of 5 dimensions: mobility, self-care, usual activities, pain/discomfort, and anxiety/depression. Each dimension consists of 5 levels: no difficulty, slight difficulty, moderate difficulty, severe difficulty, and unable to complete/extreme difficulty. The five response levels (1, 2, 3, 4, 5) correspond to each dimension in the given order. Firstly, a five level health scale was obtained (like:11111, means no difficulty in mobility, self-care, usual activities, pain/discomfort, and anxiety/depression), the EQ-5D-5L can represent a total of 3,125 (5^5^) different health states (38). Secondly, a single EQ-5D-5L value was obtained from a utility value integral system developed by Luo (39), which ranges from -0.391 to 1. 0 indicates death and 1 indicates the “perfect health” of HRQoL of an individual (40). EQ-5D-5L value less than 0 represents a health state considered worse than death (37, 41). Chinese scholars have validated the Chinese version of the EQ-5D-5L, which has demonstrated good reliability and validity. The matching process of obtaining EQ-5D-5L value can be found in the additional file (**Supplementary Tables 1**, **2**)

#### Depression

The 9-item Patient Health Questionnaire (PHQ-9) was used to measure the depressive symptomatology in the study participants. For example, participants were asked the question, “How often do you feel bored with everything or not want to do anything at all” and the respondent selects 1 answer out of 4 alternative items, including “Never (scored as 0), A couple days (scored as 1), more than half the days (scored as 2), close to every day (scored as 3)”, each item is scored from 0 to 3 as a response mechanism to measure the depressive symptomatology in the past two weeks. The total score ranges from 0 to 27, with higher scores indicating greater symptoms of depression (17). The cut off score over or equal to 10 is considered to indicate depression in accordance with previous study (2). The Chinese version of PHQ-9 has been well-validated in China (2, 41, 42) and has demonstrated good internal consistency. Additionally, due to its high sensitivity, simplicity, and ease of operation of PHQ-9 itself, most participants can complete the questionnaire within 5 min even without any assistance.

#### Covariates

Associated factors contributing to depression included in the current study were identified through a literature review about the RBICR study in China (2, 43, 44). Age was grouped into “19-30 years”, “31-40 years”, “41-50 years”, “51-59 years”, “≥60 years “; place of residence was grouped into “urban” and “rural”; household registration was grouped into “non-agriculture” and “agriculture”; education level was grouped into “primary school or below”, “junior middle school”, “high school”, “2/3 year college degree”, “undergraduate” and “postgraduate/doctor”; marital status was grouped into “unmarried”, “married”, “divorced” and “widowed”; employment status was grouped into “students”, “on a job”, “retired” and “else”; average family monthly income was grouped into “≤3000 yuan”, “3001-6000 yuan”, “6001-9000 yuan” and “≥9001yuan”; drinking frequency was grouped into “never”, “less than 1 d/month”, “1-3 d/month”, “1-2 d/week”, “3-4 d/week”, “4-5 d/week” and “everyday”. BMI was calculated by the formulas BMI= Weight(/Kg)/Height(/m)^2^ and grouped into “<24Kg/m^2^”, “24-27.99Kg/m^2^” and “≥28Kg/m^2^” (45). A total of 16 kind of diseases were investigated (30), including hypertension, stroke, coronary heart disease, dyslipidemia, Diabetes mellitus, malignant neoplasm, asthma, chronic obstructive pulmonary disease, chronic kidney disease, chronic enteritis, chronic gastritis, viral hepatitis, fatty liver disease, Alzheimer’s disease, Parkinson’s disease, mood disorders and the total number of chronic diseases were renamed multiple chronic conditions, which were grouped into “0”, “1”, “2”, and “≥3”.

### Statistical analyses

**Figure 2:**
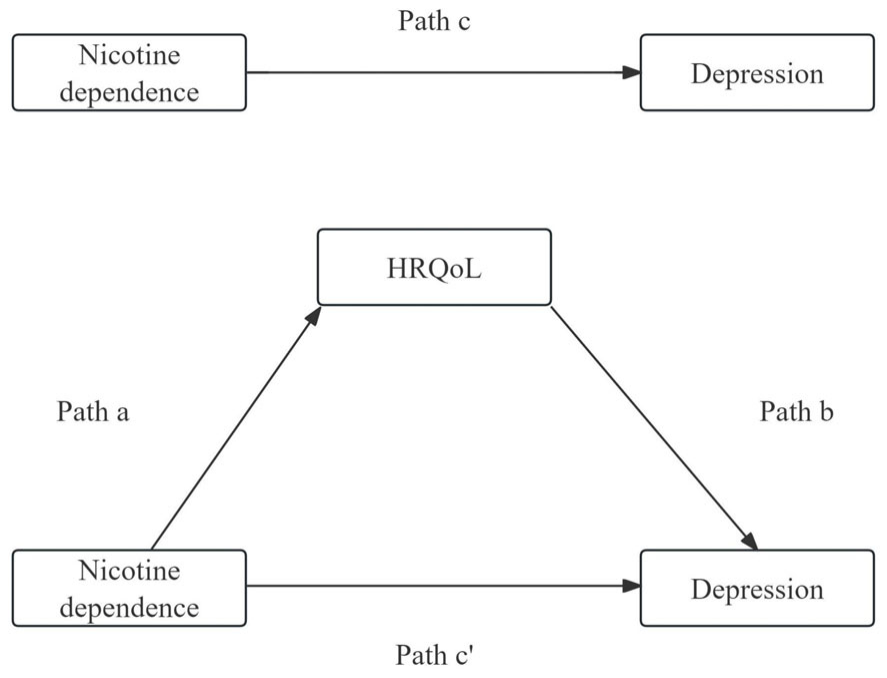
The detailed framework of mediation analysis of HRQoL on the relationship between nicotine dependence and depression.

SPSS 25.0 (SPSS, Chicago, IL, USA) was conducted for the data analysis. Numerical variables were expressed as mean ± standard deviation and the difference between two groups was conducted by independent sample t-test. Categorical variables were expressed as frequencies and percentage and frequency distributions were evaluated by chi-square (*χ^2^*) test between two groups. The associated factors (including age, gender, place of residence, household register, education level, marital status, employment status, average family monthly income, nicotine dependence, drinking frequency, living alone, BMI, multiple chronic conditions, HRQoL) contributing to depression were evaluated using logistic regression analysis, OR (odds ratio) and 95%CI (confidence interval) were calculated to demonstrate the association. Pearson’s correlation test was conducted to examine the correlation among nicotine dependence, HRQoL and depression. The reliability and validity of the measurements for HRQoL and depression were assessed using Cronbach’s α. Based on the previous studies, nicotine dependence was demonstrated to have a direct effect on HRQoL (path a), and HRQoL was demonstrated to have a direct effect on depression (path b). Thus, nicotine dependence can indirectly affect depression by the mediating effects of HRQoL, which is presented as path c’. The detailed framework of mediation analysis is presented in **Figure 2**. The mediating effect of HRQoL on the relationship between nicotine dependence and depression was assessed using Macro PROCESS 3.1 in SPSS. A bias-corrected bootstrap confidence interval was generated with model 4 after adjusting for confounding factors (using a bootstrap sample of 5,000). Considering the bidirectional relationship between smoking and depression, we also analyzed depression leading to nicotine dependence with HRQoL mediating the relationship, see **Supplementary Table 3**. It is considered statistically significant when the p-value falls below 0.05.

## Results

### Demographic characteristics of the participants

The demographic characteristics of the participants are summarized in **Table 1**. A total of 1,381 current smokers were included in the current study. Among these participants, approximately 11.95% were over 60 years old, 92.03% of the participants were males. 68.79% of the participants were resided in urban areas. 55.90% of the participants had a non-agricultural household registration, In terms of education, 25.27% of the participants had an undergraduate level of education. 74.66% of the participants were married. 55.61% of the participants were on a job. 38.67% of the participants had an average income of ¥3001-6000 yuan. Additionally, 18.03% of the participants reported never drank alcohol. 14.27% of the participants were living alone. 33.09% of the participants were in the status of overweight (24~27.99Kg/m^2^) and 7.24% of the participants were in the status of obesity (≥28Kg/m^2^). 74.44% of the participants did not suffer from chronic diseases.

**Table 1:** The demographic characteristics of the participants.

Characteristics	Total (n=1,381)		Depression				Statistical value	*P* value
			NO		YES			
Age, n/%							17.308	<0.001
19-30	316	22.88	223	70.57	93	29.43		
31-40	272	19.70	192	70.59	80	29.41		
41-50	425	30.77	348	81.88	77	18.12		
51-59	203	14.70	173	85.22	30	14.78		
≥60	165	11.95	131	79.39	34	20.61		
Gender, n/%							18.196	<0.001
Male	1271	92.03	1000	78.68	271	21.32		
Female	110	7.97	67	60.91	43	39.09		
Place of residence, n/%							1.228	0.268
Urban	950	68.79	726	76.42	224	23.58		
Rural	431	31.21	341	79.12	90	20.88		
Household register, n/%							0.201	0.654
Non-agriculture	772	55.90	593	76.81	179	23.19		
Agriculture	609	44.10	474	77.83	135	22.17		
Education level, n/%							10.428	<0.001
Primary school or below	178	12.89	134	75.28	44	24.72		
Junior middle school	250	18.10	221	88.40	29	11.60		
High school	282	20.42	211	74.82	71	25.18		
2/3 year college degree	262	18.97	208	79.39	54	20.61		
Undergraduate	349	25.27	259	74.21	90	25.79		
Postgraduate/doctor	60	4.34	34	56.67	26	43.33		
Marital status, n/%							34.114	<0.001
Unmarried	282	20.42	185	65.60	97	34.40		
Married	1031	74.66	836	81.09	195	18.91		
Divorced	39	2.82	27	69.23	12	30.77		
Widowed	29	2.10	19	65.52	10	34.48		
Employment status, n/%							46.187	<0.001
Students	124	8.98	66	53.23	58	46.77		
On a job	768	55.61	620	80.73	148	19.27		
Retired	110	7.97	85	77.27	25	22.73		
Else	379	27.44	296	78.10	83	21.90		
Average family monthly income/yuan, n/%							1.789	0.181
≤3000	379	27.44	285	75.20	94	24.80		
3001-6000	534	38.67	436	81.65	98	18.35		
6001-9000	242	17.52	185	76.45	57	23.55		
≥9001	226	16.36	161	71.24	65	28.76		
Nicotine dependence $\left(\bar{x}\pm s\right)$	1.36 ± 1.50		1.31 ± 1.47		1.51 ± 1.60		-2.063	0.043
Drinking frequency, n/%							0.202	0.653
Never	249	18.03	193	77.51	56	22.49		
Less than 1 d/month	178	12.89	130	73.03	48	26.97		
1-3 d/month	233	16.87	188	80.69	45	19.31		
1-2 d/week	286	20.71	226	79.02	60	20.98		
3-4 d/week	169	12.24	132	78.11	37	21.89		
4-5 d/week	146	10.57	110	75.34	36	24.66		
everyday	120	8.69	88	73.33	32	26.67		
Living alone, n/%							11.173	<0.001
No	1184	85.73	933	78.80	251	21.20		
Yes	197	14.27	134	68.02	63	31.98		
BMI/Kg/m^2^, n/%							0.734	0.392
<24	824	59.67	626	75.97	198	24.03		
24~27.99	457	33.09	366	80.09	91	19.91		
≥28	100	7.24	75	75.00	25	25.00		
Multiple chronic conditions, n/%							4.462	0.035
0	1028	74.44	804	78.21	224	21.79		
1	226	16.36	172	76.11	54	23.89		
2	88	6.37	68	77.27	20	22.73		
≥3	39	2.82	23	58.97	16	41.03		
HRQoL $\left(\bar{x}\pm s\right)$	0.95 ± 0.13		0.96 ± 0.08		0.88 ± 0.21		6.936	<0.001

HRQoL, Health-related quality of life.

BMI, Body mass index.

### The association between different characteristics and depression using *χ^2^*/*t* test

The association between basic characteristics and depression is presented in **Table 1**. The total prevalence of depression was 22.74% (314/1,381). The prevalence of depression was found to be statistically significant in different age, gender, education level, marital status, employment status, nicotine dependence scores, whether living alone, multiple chronic conditions and HRQoL scores.

### The association between different characteristics and depression using logit model

Subsequently, the Logit model was conducted to analyze the association between different characteristics and depression, the results are presented in **Table 2**. In the univariable regression model, nicotine dependence was positively associated with depression(OR:1.094, 95%CI: 1.008—1.187), while HRQoL was negatively associated with depression(OR:0.011, 95%CI: 0.004—0.033). In the multivariable regression model, HRQoL remained notably associated with depression(OR:0.008, 95%CI: 0.002—0.027). However, the positive association between nicotine dependence and depression was not observed.

**Table 2:** The association between different characteristics and depression using logit model.

Variables	Univariable Regression Model			Multivariable Regression Model		
	*OR*	95%*CI*	*P*	*OR*	95%*CI*	*P*
Age	0.808	(0.731—0.894)	<0.001	0.783	(0.682—0.898)	<0.001
Gender	2.368	(1.578—3.554	<0.001	1.840	(1.176—2.881)	0.008
Place of residence	0.855	(0.649—1.128)	0.268	0.895	(0.612—1.309)	0.569
Household register	0.944	(0.732—1.216)	0.654	1.084	(0.759—1.549)	0.658
Education level	1.155	(1.058—1.260)	0.001	1.071	(0.950—1.208)	0.261
Marital status	0.719	(0.566—0.914)	0.007	0.912	(0.690—1.206)	0.519
Employment status	0.855	(0.751—0.975)	0.019	0.982	(0.835—1.155)	0.827
Average family monthly income	1.087	(0.962—1.227)	0.181	1.073	(0.930—1.237)	0.336
Nicotine dependence	1.094	(1.008—1.187)	0.031	1.036	(0.948—1.132)	0.433
Drinking frequency	1.016	(0.950—1.086)	0.653	1.031	(0.959—1.109)	0.408
Living alone	1.748	(1.256—2.431)	<0.001	1.433	(0.995—2.064)	0.053
BMI	0.915	(0.746—1.121)	0.392	0.937	(0.751—1.170)	0.566
Multiple chronic conditions	1.191	(1.012—1.401)	0.035	1.182	(0.966—1.445)	0.104
HRQoL	0.011	(0.004—0.033)	<0.001	0.008	(0.002—0.027)	<0.001

HRQoL, Health-related quality of life.

BMI, Body mass index.

### Descriptive results of nicotine dependence, HRQoL, and depression scores

The descriptive results of nicotine dependence, HRQoL, and depression scores are presented in **Table 3**. The 1,381 participants showed a moderate level of nicotine dependence with a mean of 1.36(SD=1.50, range: 0-6). Approximately 9.49% (131/1,381) of the participants were classified as having heavy nicotine dependence. The participants had a relatively high level of HRQoL scores, with a mean score of 0.94 (SD=0.13, Range:-0.12-1.00). Approximately 64.73% (894/1,381) of the participants were reported being in “perfect health”. The participants’ depression score ranged from 0 to 27, with a mean of 6.48(SD=6.09). Approximately 22.74% (314/1,381) of the participants’ scores were ≥ 10, which was considered to indicate depression.

**Table 3:** The descriptive results of nicotine dependence, HRQoL, and depression scores.

Construct	Items	Range	Cronbach’s α	Statistical value	*P* value	Mean ± SD
Nicotine dependence	2	0.00-6.00	0.500	186.359	<0.001	1.36 ± 1.50
HRQoL	5	-0.12-1.00	0.793	2469.886	<0.001	0.94 ± 0.13
Depression	9	0.00-27.00	0.957	10383.818	<0.001	6.48 ± 6.09

HRQoL, Health-related quality of life.

### The correlation among nicotine dependence, HRQoL, and depression scores using Pearson’s correlation test

The correlation among nicotine dependence, HRQoL, and depression scores using Pearson’s correlation test is presented in **Table 4**. Nicotine dependence was negatively correlated with HRQoL (r_s_= -0.147, *P*<0.001), indicating that higher nicotine dependence was associated with lower HRQoL. HRQoL was negatively correlated with depression (r_s_= -0.275, *P*<0.001), indicating that lower HRQoL was associated with higher levels of depression. In contrast, nicotine dependence was positively correlated with depression (r_s_= 0.136, *P*<0.001), suggesting that higher nicotine dependence was associated with higher levels of depression.

**Table 4:** The correlation among nicotine dependence, HRQoL, and depression scores using Pearson’s correlation test.

Variables	Nicotine dependence	HRQoL	Depression
Nicotine dependence	1.000	-0.147^***^	0.136^***^
HRQoL	—	1.000	-0.275^***^
Depression_	—	—	1.000

HRQoL, Health-related quality of life.

***P<0.001.

### Mediation analysis of HRQoL on the relationship between nicotine dependence and depression

**Figure 3:**
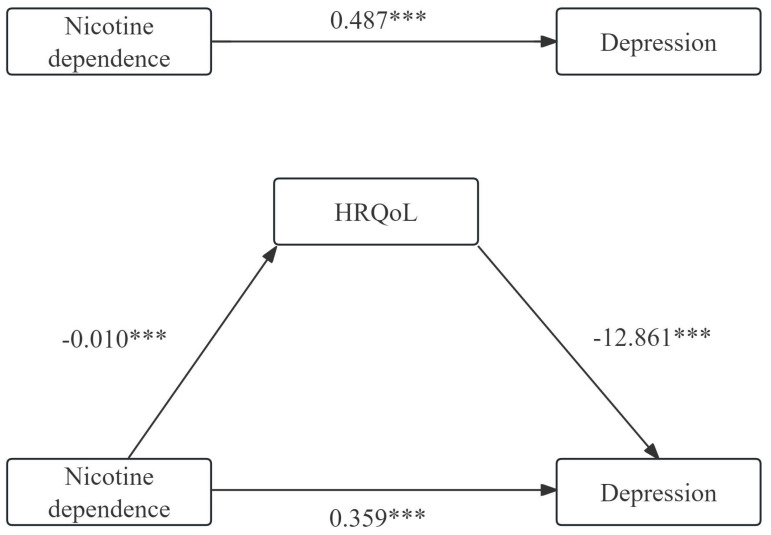
The mediating analysis of HRQoL on the relationship between nicotine dependence and depression. ***P<0.001.

The total effect, direct effect, indirect effect, and the different pathways among nicotine dependence, HRQoL and depression are presented in **Table 5**. Nicotine dependence was negatively correlated with HRQoL while positively correlated with depression. There was a negative correlation between HRQoL and depression and a mediating effect between nicotine dependence and depression mediated by HRQoL, with the proportion of the mediating effect being 26.49%. The mediated effect analysis is shown in **Figure 3**.

**Table 5:** The total effect, direct effect, indirect effect and the different pathways among nicotine dependence, HRQoL and depression.

Pathway	Index	Effect type	Effect value	95% *CI*	*S.E.*	*Statistical value*	*P value*
Nicotine dependence =>Depression	c	Total effect	0.487	0.277—0.697	0.107	4.554	<0.001
Nicotine dependence => Depression	c’	Direct effect	0.359	0.155—0.563	0.104	3.450	<0.001
Nicotine dependence => HRQoL	a	—	-0.010	-0.014 — -0.006	0.002	-4.522	<0.001
HRQoL => Depression	b	—	-12.861	-15.335 — -10.388	1.261	-10.200	<0.001
Nicotine dependence => HRQoL => Depression	a*b	Indirect effect	0.129	0.014—0.052	0.010	13.414	<0.001
Indirect effect/%			26.49				

HRQoL, Health-related quality of life.

S.E, standard error.

CI, confidence interval.

## Discussion

To our knowledge, this is the first study focusing on HRQoL involved in the relationship between nicotine dependence and depression in the general Chinese current smokers. We found that HRQoL mediated 26.49% of the association between nicotine dependence and depression in current smokers.

In the analysis, we observed that smokers with depression had higher scores of nicotine dependence and there was a positive association between nicotine dependence and depression (r_s_=0.136, *P*<0.001). Despite those findings, there was no significant association between nicotine dependence and depression (OR=1.036, 95%CI:0.948 to 1.132) in multivariable regression model. There are several reasons that could help explain this discrepancy. First, in the current study, only 9.49% of smokers in our study exhibited heavy nicotine dependence. This relatively low prevalence of heavy nicotine dependence in our sample may have limited the statistical power to detect a significant association. Second, the nicotine dependence level between participants with and without depression were relatively close, with most participants scoring below 2. This narrow range of nicotine dependence scores and lower Cronbach’s α used to evaluate the nicotine dependence level may have weakened the impact of nicotine dependence on depression, making the significant association cannot be clearly concluded from multivariable regression analysis.

Published manuscripts have suggested that smoking may be associated with an increased risk of developing depression (7, 8). A systematic review of 148 studies on the association of cigarette smoking with depression and anxiety found that smoking at baseline is associated with an increased risk of depression in later life (46). During a 6.7 years follow-up, a Korean longitudinal study found a dose dependent relationship between smoking and the risk of depression (8). It was found that 21.0% of smokers were identified as suffering from possible anxiety or depression (47). Nicotine may enhance the intensity and duration of positive emotions and self-perceived energy (48), and may also reduce depressive symptoms by aiding in mood regulation (49).

There is a complicated and reciprocal relationship between smoking and depression. In 1998, Escobedo et al. found that depressive symptom or depression predicted the initiation of smoking (50). The prevalence of smoking is higher in depressed patients than in non-depressed patients, but the success rate of smoking cessation is lower than in non-depressed patients (51). Smoking behavior can predict the risk of depression, and depression also can predict the onset of smoking behavior. Additionally, there was an interaction effect between depressive symptoms and total daily cigarettes consumption in heavy smokers (1). According to study of Wootton et al., smoking was a risk factor for depression and Pasco (10) et al. reported that tobacco smoking doubled the risk of developing major depression in females followed up for 10 years (52).

Nicotine dependence has been found to increase the risk of depression. Nicotine dependent smokers exhibit more severe symptoms of depression and/or anxiety compared to smokers without nicotine dependence, former smokers, and never smokers (53). Sung et al. demonstrated that smoking amount was associated with the risk of depressive symptoms (8). Prolonged smoking can lead to nicotine dependence, which is associated with several common neurobiological mechanisms underlying depression (54). Previous studies have found that depressed mood was positively correlated with tobacco dependence, the higher of tobacco dependence, the more severe of depressive mood (55, 56). Smoking can provide temporary relief from depression by stimulating the nervous system (57). While prolonged smoking can lead to nicotine dependence, which increases susceptibility to develop depression (49).

The mechanisms behind nicotine dependence affected depression are likely to be complex, involving biogenetic, psychological factors. As a result of nicotine activating the nicotinic acetylcholine receptors (nACHRs), increases the release of neurotransmitters, such as dopamine, 5-hydroxytryptophan, and γ-aminobutyric acid, this leads to a sense of excitement and pleasure, reducing anxiety and tension (12, 51). Nicotine dependence was mediated by dopaminergic reward pathways and dopamine dysfunction could drive depression (58). For another angle, smokers rely on smoking to cope with negative emotions. On the other hand, when they quit, they may experience withdrawal symptoms, including negative emotions, which can lead to a relapse in smoking. To alleviate these negative emotions, individuals who have quit smoking may resume smoking behavior. Therefore, addressing negative emotions in smokers is more conducive to successful smoking cessation.

Previous studies have demonstrated that smoking was negatively correlated with lower HRQoL (59–61). In the current study, nicotine dependence was negatively associated with poor HRQoL. Smokers with light nicotine dependence had a higher score of HRQoL than those with moderate and heavy nicotine dependence smokers. This may be that nicotine dependent smokers experience barriers to mental health, which affect their subjective assessment of HRQoL. Thus, the current study highlights the differences among smokers with varying levels of nicotine dependence and calls for exploration of the association between nicotine dependence and HRQoL. This is important for raising social awareness about the health concerns of smokers and improving HRQoL among those smokers with nicotine dependence.

The present study conducted a bootstrap mediation analysis to partially verify the hypothesis that HRQoL affected as an indirect factor in the correlation between nicotine dependence and HRQoL. The results demonstrated that HRQoL mediated 26.49% of the association between nicotine dependence and depression in current smokers, indicating that HRQoL played a mediating role. However, longitudinal studies are required to confirm this mediating relationship.

Our study provides deeper and additional insight into the association between smoking and depression from a mediation perspective mediated by HRQoL. From a practice perspective, we recommend that professionals, such as public health agencies, policymakers, and community members, pay more attention to smokers with nicotine dependence in order to inform the smokers of the effects of smoking on depression mediated by HRQoL. When starting treatment for depression in smokers, attention should be considered to increase the smoker’s physical state, mental function, social competence, and overall personal condition. The current study has the following limitations. First, the causal relationship between nicotine dependence, HRQoL and depression could not be determined with a cross-sectional study. Second, nicotine dependence, HRQoL and depression were measured by self-reported measures from the study participants, which might be underestimated or overestimated by recall or social expectation bias. Third, we only considered depression; other negative emotions, including, anxiety, anger, panic and etc. were not included in the current study. Future research should consider these negative emotions. Fourth, the measure of PHQ-9 in depression and EQ-5D-5L in HRQoL are partly overlapped in depressive symptoms. Whether this overlap will affect the conclusions is unknown, so we recommend that future researchers should realize this overlap between HRQoL and depression measure. Finally, the relationship among nicotine dependence, HRQoL and depression are complicated and reciprocal, and we have only explored their relationship from the perspective of mediation analysis in 1,381 participants. Considering that China’s huge nicotine dependence population, which is also a limitation for the results to extrapolate, cohort studies with large samples are needed to support our conclusions.

## Conclusions

The current study demonstrates that nicotine dependence is negatively correlated with HRQoL while positively correlated with depression and HRQoL is negatively associated with depression in current smokers. HRQoL mediated the relationship between nicotine dependence and depression. Based on the results of our study, these findings can help develop interventions to improve quality of life in current smokers to alleviate depression.

## Data Availability

The original contributions presented in the study are included in the article/**Supplementary Material**. Further inquiries can be directed to the corresponding author.
