# Supplementary material for: Correlations among nicotine dependence, health-related quality of life, and depression in current smokers: a cross-sectional study with a mediation model
Source: Front Psychiatry. 2024 Aug 27;15:1455918. doi: 10.3389/fpsyt.2024.1455918 (PMC11384568; doi:10.3389/fpsyt.2024.1455918)
Supplement: Supplementary file 1 [file DataSheet1.zip › Data sheet 1/Supplementary table 3.doc]

Supplementary table 3

| The total effect, direct effect, indirect effect and the different pathways among depression, HRQoL and nicotine dependence | | | | | | | |
| --- | --- | --- | --- | --- | --- | --- | --- |
| Pathway | Index | Effect type | Effect value | 95% CI | *S.E.* | *t* | *P* |
|  |  |  |  |  |  |  |  |
| Depression=>Nicotine dependence | c | Total effect | 0.031 | 0.017—0.044 | 0.007 | 4.553 | ＜0.001 |
| Depression=>Nicotine dependence | c' | Direct effect | 0.024 | 0.010—0.038 | 0.007 | 3.449 | 0.001 |
| Depression=>HRQoL | a | — | -0.006 | -0.007—-0.005 | 0.001 | -10.657 | ＜0.001 |
| HRQoL=>Nicotine dependence | b | — | -1.149 | -1.810—-0.487 | 0.338 | -3.404 | 0.001 |
| Depression =>HRQoL=>Nicotine dependence | a*b | Indirect effect | 0.007 | 0.008—0.050 | 0.010 | 0.638 | 0.524 |
| indirect effect/% |  |  | 21.47% |  |  |  |  |
